# Exploring Experiences of Conflict within Medical Teams in an Emergency Department: A Focus Group Approach during the COVID-19 Pandemic

**DOI:** 10.3390/healthcare12070727

**Published:** 2024-03-26

**Authors:** Wen-Chih Fann, Chih-Mimng Hsu, Cheng-Ting Hsiao, Bih-O Lee

**Affiliations:** 1 Department of Emergency Medicine, Chang Gung Memorial Hospital, Chiayi 613, Taiwan; patrickfann@cgmh.org.tw (W.-C.F.); qcth3160@cgmh.org.tw (C.-T.H.); 2 Medical Education Department, Chang Gung Memorial Hospital, Chiayi 613, Taiwan; kan200068@cgmh.org.com; 3 Department of Business Administration, College of Management, National Chung Cheng University, Minhsiung, Chiayi 621301, Taiwan; 4 School of Medicine, College of Medicine, Chang Gung University, Taoyuan 33302, Taiwan; 5 School of Nursing, College of Nursing, Kaohsiung Medical University, Kaohsiung 80708, Taiwan; 6 Center for Innovative Research on Aging Society (CIRAS), National Chung Cheng University, Chiayi 621301, Taiwan

**Keywords:** emergency department, conflict experience, focus group

## Abstract

The factors related to conflicts in emergency departments (EDs) have been studied for decades. The post-pandemic digital era may transform the medical landscape in EDs, potentially changing the patterns of conflict between healthcare professionals. This study used focus group interviews to explore conflicts in EDs. Four groups, each with 4–6 participants, took part in this study. Semi-structured interviews were conducted using six research questions. Summative content analysis was used to analyze the data. The participant’s average age was 37.82 years, and the average number of working years was 12.12. The following five themes emerged: multiple patterns of internal conflict; external conflicts arising from cross-departmental coordination; conflicts due to unclear job boundaries; adapting to conflicts in diverse ways; and seeking hospital arbitration. The results of this study suggest extending interdisciplinary collaborative practice from emergency departments to all coordinating departments. An inclusive environment for equality between professions and open communication should be promoted by hospitals.

## 1. Introduction

Emergency care systems are responsible for organizing and facilitating access to critical medical care, both during transportation and within healthcare facilities, in order to save lives [1]. The fast-paced nature of emergency departments (EDs) can contribute to conflicts due to time pressures and high levels of stress. After conflicts, ED professionals may experience frustration, burnout, or emotional exhaustion, leading to further conflicts with colleagues [2]. 

Conflicts between healthcare professionals can arise due to several factors. Misunderstandings or lack of effective communication between medical teams can lead to conflicts. In addition, conflicts can arise due to the ambiguity of roles—a lack of clarity regarding roles and responsibilities. Healthcare professionals may have different perspectives on patient care that can lead to conflicts. Furthermore, conflicts can arise due to staffing challenges, such as inadequate staffing levels, workload imbalance, or scheduling conflicts [3]. A recent study indicated that the causes of conflicts in medical teams included excessive workload, excessive resource use, inconsistent assessments, and limited resources [4]. 

Hierarchical conflicts may occur between healthcare professionals of different hierarchical positions. Such conflicts may arise within ED medical teams due to differences in professional hierarchies and power dynamics [2,3], for example, conflicts between physicians and nurses and between senior and junior staff members [2]. Several effective interventions, such as staff training programs, patient education initiatives, and the use of security personnel, may be used to reduce hierarchical conflicts [5].

The boundaries of healthcare professional roles may not be clearly defined. For example, the relationships between advanced practice nurses (APNs) and nurses have rarely been explored in light of theories on boundary work and professional identity. To avoid the risk of being restricted to collaborations with physicians, De Rosis et al. (2023) indicated how pre-APNs engage in various forms of boundary work with nurses to negotiate new professional relationships with their colleagues [6]. Chan et al. (2014) explored the aspects of internal conflict in the ED. The study identified 12 negative conflict patterns and 10 themes to avoid conflict escalation [7]. It mentioned that conflicts among healthcare professionals often arise due to differing perspectives on patient care plans. To clarify professional boundaries and responsibilities, the coordination service within a hospital is crucial. Further, the successful coordination of services, such as conflict management in this institution, may reduce conflicts between healthcare professionals [8].

Research on factors related to ED conflicts has been conducted for more than 10 years. The COVID-19 pandemic and digital transformation may alter the medical and healthcare landscape in EDs, potentially changing the patterns of conflicts between healthcare professionals. A recent study interviewed ED physicians from two hospitals to explore their moral distress during the COVID-19 pandemic. The study found that ED physicians experienced more moral distress during the COVID-19 pandemic, particularly in situations of resource scarcity, hospital bed shortage, and strict triage protocols, where they had to rigorously navigate their moral boundaries [9]. Although this current study might be not directly related to conflicts in the ED, it suggests that during a pandemic, physicians may have better control over medical ethics to ensure the safety of healthcare workers and patients and potentially reduce medical conflicts. Further research is still needed to validate these findings. Therefore, this study aimed to apply focus group interviews to understand the sources of conflicts in an ED medical team, identify barriers to conflict resolution and explore the experience of using strategies to resolve conflicts in ED medical teams.

## 2. Methods

### 2.1. Design

This study used focus group interviews to explore conflicts within ED medical teams.

### 2.2. Settings and Participants

This study was conducted in the ED of a regional teaching hospital that had over 1000 beds. The ED consisted of 25 physicians and 48 nurses and nurse practitioners (NPs). Purposive sampling was used to invite medical professionals from the ED to take part in focus groups. In this study, there were four focus groups, each with four to six participants [10]. The focus group participant composition was based on homogeneity due to their similar backgrounds.

### 2.3. Data Collection

This study was initiated before the COVID-19 pandemic and conducted during the period of COVID-19. The data collection period extended from April 2022 to March 2023. The research team invited all medical and nursing staff in the ED to participate through individual email invitations. The participants did not know in advance who else was going to participate. The interviewer of the focus group was a female nursing faculty member with a PhD and experience in conducting interviews. She has worked with the ED as an academic consultant for several years. A male medical doctor observed participants’ reactions and reminded them of the interview procedures [10].

Before each interview, the interviewer explained the purpose of the study and completed the consent form with all participants. Each participant was given a paper copy of the research questions, and a large poster was placed on the board in the meeting room to facilitate the focus group interviews. Each focus group interview was audio-recorded. As no new themes emerged after four focus group interviews were completed, the research team deemed that data saturation had been achieved and decided to stop data collection.

The focus group interviews were conducted as semi-structured interviews. The interview questions were reviewed and revised by two ED experts before being officially used. The interviews took place in a hospital meeting room that could accommodate 20–30 people. Each group interview took 60–90 min. The interview questions were as follows. (a) How would you describe your experience with ED conflicts? (b) Can you share any specific incidents or examples related to ED conflicts? (c) What are your thoughts on specific aspects of ED conflicts? (d) How do you perceive the impact of ED conflicts on relevant stakeholders? (e) Are there any challenges or barriers you have encountered with ED conflicts? (f) Can you suggest potential solutions to ED conflicts?

### 2.4. Rigor

This study adhered to the four trustworthiness criteria proposed by Lincoln and Guba (1985) [11]. (a) Credibility: The interviewer had a background in emergency nursing that enabled her to gain the trust of participants and facilitated the sharing of participants’ authentic experiences. The research team discussed the findings four times and invited two experts to examine the accuracy of the analysis. (b) Transferability: the rich experiences of ED professionals obtained from the interviews allowed the research findings to be applied to other clinical contexts. (c) Dependability: the transcripts were personally organized and analyzed by the interviewer and moderator of the focus group interviews. The research team continually reviewed the audio recordings and the written transcripts of each meeting. (d) Confirmability: all the original data and content of the analysis are properly preserved for future audits.

### 2.5. Data Analysis

This study used summative content analysis that involved counting and comparing, followed by the interpretation of the underlying context [12,13]. Two coders analyzed the data separately but held meetings to reach a consensus. The analytical process included multiple careful readings of the text to gain a comprehensive understanding of the interview content. Repetitive descriptions of experiences of conflict in the ED were marked, and meaningful sentences were carefully identified from the marked content. Sentences with similar characteristics and meanings were grouped to form several themes, each of which was given a definition. All themes were integrated to establish a comprehensive exploration of conflict experiences in the ED. The themes were shared with the participants for comments and corrections.

### 2.6. Ethical Consideration

This study commenced after it was approved by the Institutional Review Board (IRB) of the study hospital (IRB no 202200418B0). The participants were informed that the interview content was treated anonymously, and they could request to stop the recording or withdraw at any time during the study.

## 3. Findings

In this study, four focus group interviews were conducted, with a total of 17 participants—10 females and 7 males. The participants included five attending physicians, three NPs, and nine nurses. The average age was 37.82 years, and the average years of work in the ED was 12.12. Five themes emerged from this study.

Theme 1: Multiple patterns of internal conflict

The ED is a setting for acute medical care, where diverse conflicts can occur between healthcare professionals. The main personnel involved in ED care are physicians, NPs, and general nurses. Therefore, conflicts between these three categories of healthcare professionals are quite common, often arising from patient management. In addition, conflicts can also be triggered by administrative regulations and policies.

Participant No. 4 (a nurse) said: “I have clashed with a physician who was undergoing postgraduate-year training. The order he issued was incorrect, so I confronted him about it, but he refused to admit his mistake and complained to the attending physician that the nurse failed to remind him. If it were a new nurse who did not notice the doctor’s incorrect order, then the patient’s safety would have been jeopardized”.

Participant No. 12 (a physician) said: “I have butted heads with a head nurse before. Most head nurses focus on the matter and not the person, but there are some who take things personally. I once got cut by an emergency board and confronted the head nurse, but they told me not to bother them about these trivial matters when COVID-19 prevention measures are ongoing. We had a heated argument afterwards”.

Participant No. 12 (an NP) said: “Some special medications for emergencies can be borrowed from the nearest pharmacy. I sent the nurse from the previous shift to borrow them, but they came back and said that the head nurse refused to lend us the medications, and the head nurse’s attitude was so terrible that it nearly triggered a conflict”.

Theme 2: External conflicts arising from cross-departmental coordination

During the process of providing care to patients in the ED, external conflicts often occur with supporting units such as the laboratory department, radiology department, and the units responsible for transferring patients to other wards. Nurses are primarily responsible for coordinating across departments. They are more prone to experiencing external conflicts.

Participant No. 2 (a nurse) said: “I encountered a critical patient before who had to cut the queue for a radiological examination. The radiology department’s perception of critical cases is different from ours. We called them beforehand about the emergency case, but by the time I arrived there with the patient, we were locked outside. Nurses focus on the patient, but the radiology department focuses on examinations instead”.

Participant No. 3 (a nurse) said: “Delayed treatment occurs when emergency care assistants fail to send patients for an examination in a timely manner or fail to deliver the samples and reports to the physician. This could lead to a brain hemorrhage. I worry about medical negligence and endangering patient safety”.

Participant No. 14 (a nurse) said: “I scheduled a shift handover with the ward, but the ward would delay it by three to four hours. When I pressed them about it, they would blame it on the emergency services and question the urge to handover. Conflicts happen when they always tend to emergency patients”.

Theme 3: Conflicts due to unclear job boundaries

In Taiwan, there is some overlap in the roles and responsibilities of physicians and NPs, especially in some medical management areas. Due to unclear job responsibilities, ambiguous accountability, and longstanding grievances, conflicts often arise between physicians and NPs. In Taiwan, physicians hold more authority in the healthcare system. When conflicts arise between physicians and NPs, NPs often find themselves in a relatively disadvantaged position. They may express their concerns passively and may experience negative emotions as a result.

Participant No. 7 (an NP) said: “I remember a conflict that happened a long time ago. The physician in question was no longer a supervisor, but he still had a bone to pick with me. I was mentally traumatized and unable to vindicate myself. The supervisor was sitting on the fence as well. Afterwards, I became passive in participating or helping out at the ED”.

Participant No. 8 (an NP) said: “Triages are unforeseeable. Sometimes, the patients just keep coming, and we are all tied up. I could not contain my anger and asked the physician why they were so ineffective that day. Some of the attending physicians would blast us off with foul words, especially those who tend to cuss more often. Even when we finish the job together, if we happen to make eye contact, they treat me like a criminal. I wouldn’t cuss them back in response, though”.

Theme 4: Adapting to conflicts in diverse ways

Most healthcare professionals, driven by their duty to save lives, believe that the well-being of the patient should be the first priority. They strive to avoid situations where patients or their family members feel embarrassed or recorded during medical consultations. Consequently, many professionals choose to endure conflicts silently. However, these conflicts can leave a lasting psychological impact on healthcare workers, making it difficult to find a resolution. Some adopt a positive mindset and actively seek ways to prevent conflicts from arising. They also focus on finding effective methods to cope with the unpleasant aftermath of conflicts.

Participant No. 1 (a nurse) said: “I prioritize patient treatment and resolving their issues as soon as possible. Because we are all busy, some physicians may rudely raise doubts about us, which we can only swallow begrudgingly. We have to contain our wrath and emotions because we don’t want to fight with them on the spot. The ED is an open area, so any conflicts may be captured on camera by the public. We really need to control our emotions in these situations”.

Participant No. 12 (a physician) said: “I used to clash with the ED staff most of the time. Once, I read a book that said that interpersonal interactions are like deposits; if you are friendly to another person, you will remember the encounter. If another interaction later is unpleasant, the experience can easily be offset or forgotten because of the previous friendly encounter. So, it’s very important to handle conflicts in a positive light. All of us were covered in personal protective equipment during the pandemic, and we seldom had time to chat. These good interactions are often overlooked”.

Theme 5: Seeking hospital arbitration

Over half of the participants mentioned that if a serious conflict occurs in the ED, causing patient safety issues, the ED head nurse and ED physician director are not able to handle it after reporting. The conflicts may be reported to the hospital for investigation and arbitration. In this study, physicians were more willing to describe the process of reporting upwards, while nurses and NPs only mentioned that they could report upwards.

Participant No. 11 (a physician) said: “In emergencies, everyone needs to maintain their composure. We refer healthcare workers, patients, or anyone that might cause a conflict to the hospital’s healthcare quality and patient safety committee for arbitration and management in order to guarantee patient safety”.

Participant No. 16 (a physician) said: “If we are unable to resolve a conflict on the spot, we usually report it to the head nurse or ED director. If the conflict is so severe that it endangers patient safety or involves other units, then we directly report it to the senior hospital management because the conflict may be beyond the ED’s scope of management”.

## 4. Discussion

This study explores the experiences of conflict in the ED. While some of the findings may be similar to those of previous studies, they hold significance in the context of the modern era and can contribute to the development of new ideas for medical care in EDs.

The first theme emerging from this study is related to internal conflicts and suggests that conflicts most commonly occur between physicians and nurses, as they are the frontline healthcare professionals providing direct care to patients [2]. This study was conducted during the COVID-19 period, and the increased workload related to COVID-19 might have led to more conflicts. This study also showed that internal administrative regulations hindered efficient ED functioning, for example, the ability to obtain urgent medication from the ED pharmacy. This suggests the need to accelerate the digital transformation of ED administration.

The second theme is related to external conflicts that arose from problems with communication with different departments in the hospital. Nurses faced more external conflicts because they needed to coordinate with other departments in the hospital. Each department prioritized its own tasks without considering the urgency of the ED patients. It was clear that nurses had a care philosophy that was more patient-centered, as mentioned in a previous study [3]. Issues with patient safety should be the first priority of ED medical care. These external conflicts may be reduced through communication between department managers and the digitalization of the hospital system.

The notable aspect of the third theme was the overlap of responsibilities between physicians and NPs. This finding is similar to those of previous studies [2,3,5]. In this study, conflicts between physicians and NPs arose mostly in relation to medical treatments. Although the boundaries of professional roles have been clearly defined in national regulations [14], the boundaries between the professional roles of physicians and NPs in EDs may not be easy to demarcate. Since NPs are trained to perform some of the work of resident physicians, the enhancement of interdisciplinary collaborative practices (IPPs), such as communication and collaboration, may help to reduce conflicts in EDs [15].

The fourth theme shows that ED professionals may adapt to conflicts through positive ways of coping. Some conflicts cast a long-lasting shadow and diminish the professionals’ enthusiasm for work. ED managers should be aware of the institutional culture and its influence on negative effects arising from ED conflicts and create an inclusive environment for ED staff [16].

The final theme indicated that when conflicts in the ED could not be resolved internally, they were reported to the hospital’s Medical Quality and Patient Safety Committee for arbitration. In Chinese culture, handling conflicts in a low-profile manner is a common approach. A unique aspect of this study was that the physicians could explain conflicts clearly and in greater detail than the other healthcare professionals. In Taiwan, an ED is normally assigned one head nurse and one physician director. The physician director may capitalize on their social advantage to work collaboratively with the head nurse to reduce conflicts in the ED. Instances of seeking hospital arbitration should be reduced under an inclusive environment.

Chan et al.’s (2014) study on internal conflicts in the ED found that the conflicts within the ED were diverse, particularly between physicians and NPs or nurses [7]. However, in real clinical situations, conflicts can be more easily resolved when physicians and NPs communicate directly, while nurses often receive second-hand information, which can lead to conflicts with consulting physicians. Nevertheless, the participants in this study did not express conflicts within the ED clearly, which may be related to the study being conducted during the COVID-19 pandemic. Another possibility is that this study was conducted in the Chinese cultural context, where people may be reluctant to express the true nature of internal conflicts in group interviews, especially since nurses have a relatively low status in the medical team. Future research can further verify these findings.

In summary, the findings of this study showed that the conflicts in the ED were diverse and complex. ED staff rarely complained about heavy workloads or insufficient resources causing conflicts, unlike in previous studies [4]. This may be because we conducted this study during the COVID-19 pandemic when fewer people sought medical care from the ED. Furthermore, during the pandemic, there was a lack of resources, and healthcare professionals might have shown a stronger spirit of solidarity; these reasons could have led to fewer conflicts. Therefore, the research findings from this study might correspond with the recent study [9], confirming that healthcare providers may increase their moral distress during a pandemic and strive to minimize medical conflicts.

### Limitations

This study was carried out during the different stages of the COVID-19 pandemic period, which may limit the generalizability of its findings. In addition, this study was conducted within the context of Chinese culture, where physicians have greater authority than nurses and NPs. The specific conflicts and coping experiences addressed in this study may also limit the applicability of the findings to other contexts.

## 5. Implications for Clinical Practice

To provide effective solutions for conflicts in the emergency department based on this study’s results, we offer the following suggestions as references for clinical practices. First, IPP in the ED traditionally includes physicians and paramedics. To reduce external conflicts, IPP may be extended from the ED to all coordinating departments, such as radiology and pharmacy. Second, there needs to be awareness of the difference in hierarchy between physicians and nurses or NPs in the extended IPP context. Furthermore, the creation of an inclusive environment to ensure equality between ED professionals and open communication should be promoted by the hospital. Lastly, the digital transformation of ED medical services needs to be accelerated, especially the use of digital management to facilitate internal and external communication, which may reduce conflicts.

## 6. Conclusions

This study found that nurses and NPs tended to be more tolerant of conflicts with physicians. The ED conflicts were more likely to be related to poor communication between administration systems in the hospital. IPP should be extended from the ED to all coordinating departments to reduce external conflicts. The creation of an inclusive environment to ensure equality between professions should be promoted. The digital transformation of the ED may facilitate internal and external communication and reduce conflicts.

## Data Availability

Data are contained within the article.
